# Two new species and a new name of *Entoloma* subgenus *Cubospora* (*Entolomataceae*, *Agaricales*) from subtropical East China

**DOI:** 10.3897/mycokeys.132.195294

**Published:** 2026-05-14

**Authors:** Yu-Qin Xu, Hui Zeng, Sheng-Nan Wang, Jun-Qing Yan

**Affiliations:** 1 Jiangxi Provincial Key Laboratory of Excavation and Utilization of Agricultural Microorganisms, Jiangxi Agricultural University, Nanchang 330045, China Jiangxi Provincial Key Laboratory of Excavation and Utilization of Agricultural Microorganisms, Jiangxi Agricultural University Nanchang China; 2 Institute of Edible Mushroom, Fujian Academy of Agricultural Sciences, Fuzhou 350011, China Jiangxi Provincial Key Laboratory of Subtropical Forest Resource Cultivation, College of Forestry, Jiangxi Agricultural University Nanchang China; 3 Jiangxi Provincial Key Laboratory of Subtropical Forest Resource Cultivation, College of Forestry, Jiangxi Agricultural University, Nanchang 330045, China Institute of Edible Mushroom, Fujian Academy of Agricultural Sciences Fuzhou China

**Keywords:** *
Basidiomycetes
*, cuboid spores, new taxa, phylogeny, taxonomy

## Abstract

Based on morphological and phylogenetic evidence, two new species of *Entoloma* subgen. *Cubospora*, *E. fucatum* and *E. pseudobrunneosquamulosum*, are described from subtropical regions of China. *Entoloma fucatum* is distinguished by its grayish-ruby pileus that is hygrophanous and striationless, cuboid basidiospores, sterile lamellar edges, and the presence of both cheilocystidia and pleurocystidia. *Entoloma pseudobrunneosquamulosum* is characterized by its collybioid basidiomata, a brown pileus with squamules on its surface and lacking striations, cuboid basidiospores, heterogeneous lamellar edges, the presence of both cheilocystidia and pleurocystidia, and pileipellis cells containing light brown intracellular pigments. Detailed descriptions, color photographs, and scanning electron micrographs of spores are presented. In addition, *E. pseudotomentosum* nom. nov. is herein proposed as a replacement name for *E. tomentosum* J.Q.Yan, L.G.Chen & S.N.Wang, which is a later homonym of *E. tomentosum* Z.S.Bi.

## Introduction

*Entoloma* (Fr.) P.Kumm., typified by *E. sinuatum* (Bull.) P.Kumm., was established by Kummer (1871). Species of *Entoloma* are globally distributed and abundant in both temperate and tropical regions, with some species even extending into alpine and frigid zones (Largent 1977; Romagnesi and Gilles 1979; Horak 1980; Baroni 1981; Noordeloos 1992; Largent 1994; Noordeloos 2004; Gates and Noordeloos 2007; Noordeloos and Hausknecht 2007; Horak 2008; Gates et al. 2009; Noordeloos and Gates 2009; Noordeloos and Gates 2012; Karstedt et al. 2024), the majority being saprotrophic in shaded, moist habitats or among mosses in forests (Noordeloos 1981; Largent 1994). To date, over 2,000 species have been described worldwide (Co-David et al. 2009; Noordeloos et al. 2018). The main characteristics of this genus include pink spore prints, angular basidiospores in all views, and remarkable morphological diversity, such as fruiting bodies exhibiting mycenoid, collybioid, omphalioid, clitocyboid, and pleurotoid forms (Co-David et al. 2009; Xu et al. 2025).

In *Entolomasensu lato*, some species have uniquely shaped basidiospores that are cuboid, with six more or less equal quadrilateral faces and a dihedral angle. Species with cuboid-shaped basidiospores are relatively easy to identify. However, historically, there have been divergent taxonomic treatments for species exhibiting this morphological trait, with different subgenera or sections being delineated solely on the basis of this characteristic, such as sect. *Staurospora* within subg. *Nolanea* or subg. *Inocephalus* (Noordeloos 1992; Largent 1994). Recently, Karstedt et al. (2019) conducted research on phylogeny and basidiospore morphology and found that the species classified based on this morphological characteristic belong to two independent subgenera: *Entoloma* subgen. *Cubospora* Karstedt, Capelari, Largent, T.J.Baroni & Bergemann, with the type species *E. luteolamellatum* (Largent & Aime) Blanco-Dios; and *Entoloma* subgen. *Cuboeccilia* Karstedt, Capelari & Largent, with the type species *E. omphalinoides* (Largent) Blanco-Dios (Karstedt et al. 2019). Species in *Entoloma* subg. *Cubospora* possess mycenoid, collybioid, or tricholomatoid habits, often have cylindro-clavate or clavate cheilocystidia, and have a pileipellis as a trichoderm, a trichoderm in transition to a cutis, or a cutis. *Entoloma* subg. *Cuboeccilia* includes species with omphalinoid or collybioid basidiomes with an umbilicate or slightly depressed pileus, adnate, emarginate-decurrent, or decurrent lamellae, and fusoid cystidia.

In China, research on *Entoloma* species has progressed rapidly, with approximately 200 species documented to date. Approximately 30 of these have cuboid spores, of which 19 have been formally described as new species (He et al. 2015; Chen et al. 2024; Liu et al. 2025). Through investigations of macrofungal diversity in subtropical regions of East China, two new species belonging to subgen. *Cubospora* were identified. These are described in detail based on comparative morphology and phylogenetic analyses.

## Materials and methods

### Morphological studies

The specimens involved in this study were gathered from Zhejiang Province, China, between 2020 and 2021 and were preserved as dried specimens. All of these specimens are housed in the Herbarium of Fungi at Jiangxi Agricultural University (HFJAU). Fresh specimens were photographed and macroscopically documented in the field. Color notations were in accordance with the Methuen Handbook of Colour (Kornerup and Wanscher 1978). Microscopic morphological structures were observed and measured under an Olympus BX53 microscope (Olympus Corporation, Tokyo, Japan). This was achieved by making squash preparations from sections of the dried specimens, which were placed in either a 5% KOH solution or water. A 1% Congo red solution was used as the staining agent for observing colorless tissues. The amyloidity of the spores was tested using Melzer’s reagent (Horak 2005; Chen et al. 2024). For each collection, the dimensions of at least 40 basidiospores, basidia, and cystidia were measured. The size range of the spores is presented in the format (*a*) *b–c* (*d)*, where “*a*” and “*d*” represent the minimum and maximum values, respectively, and 90% of the spores fall within the range of “*b–c*.” The meanings of other spore characteristics are as follows: “*Q*” represents the length-to-width ratio; “*av*” indicates the average value; numbers in square brackets [*X/Y/Z*] denote *X* basidiospores measured in *Y* basidiomata from *Z* collections; and “*Qm*” represents the average “*Q*” value ± standard deviation (Yu et al. 2020). The morphological descriptions are based on the work of Noordeloos et al. (2022). The morphology of the spores was further confirmed using an electron microscope. Specifically, a portion of the gills from the dried fruiting body was sampled and observed under an electron microscope (JEOL JSM-IT800 Schottky Field Emission Scanning Electron Microscope).

### DNA extraction, PCR amplification, and sequencing

Genomic DNA was extracted from dried specimens with the NuClean Plant Genomic DNA kit (CWBIO, China) (Wang et al. 2022). The ITS, LSU, *tef*1-α, and *rpb*2 regions were amplified using the primer pairs ITS1F/ITS4, LR0R/LR5 (Hopple and Vilgalys 1999), EF983F/EF1953R (Co-David et al. 2009), and *rpb*2-i6f-RhoF1/*rpb*2-RhoR1 (Kluting et al. 2014), respectively.

PCR amplification was conducted using a 25 µL reaction system as follows: 1 µL of DNA, 1 µL of each of the forward and reverse primers, 9.5 µL of ddH_2_O, and 12.5 µL of 2× TaqMaster Mix [Qing Ke Biotechnology Co. Ltd. (Wuhan City, China)]. PCR was performed using a touchdown program for all regions: initial 95 °C for 5 min, then 14 cycles of denaturing at 95 °C for 30 s, annealing at 65 °C for 45 s (with a decrease of 1 °C per cycle), extension at 72 °C for 1 min, then 30 cycles of denaturing at 95 °C for 30 s, annealing at 52 °C for 30 s, and extension at 72 °C for 1 min, with the final extension at 72 °C for 10 min (Bau and Yan 2021). The PCR products were sequenced by Qing Ke Biotechnology Co. Ltd. (Wuhan City, China).

### Alignment and phylogenetic analyses

Based on previous studies by Karstedt et al. (2019) and Morozova and Pham (2023), this study constructed phylogenetic trees using Bayesian inference (BI) and maximum likelihood (ML) methods, respectively, with the combined datasets of ITS + LSU + *tef*1-α + *rpb*2. Based on the research by Chen et al. (2024) and Liu et al. (2025), several species from subgenus *Nolanea* were designated as outgroups. Information on specimens and GenBank accession numbers is provided in Table 1. The ITS, LSU, *tef*1-α, and *rpb*2 sequence datasets were aligned separately on the MAFFT online server (Katoh et al. 2019). The processed sequences were subjected to BI and ML phylogenetic analyses using MrBayes v.3.2.7a and IQ-TREE v.2.1.2 software (Nguyen et al. 2015), respectively. The best-fit models of ML and BI were determined by PartitionFinder (Kalyaanamoorthy et al. 2017), complying with the corrected Akaike information criterion (AICc). For the BI analysis, the gene chains were run for 1 million generations. The first 25% of trees were discarded as burn-in. The branches of Bayesian posterior probability (BI-PP) ≥ 0.95 and ML ultrafast bootstrap proportions (UFBoot) ≥ 95% were considered statistically supported (Nguyen et al. 2015). According to the criteria for new species identification proposed by Dettman et al., a new species must exhibit one to two stable morphological differences from similar species and form independent and stable clades in the phylogenetic tree (Dettman et al. 2003).

**Table 1.** Details of sequences used in the phylogenetic analyses. Newly generated sequences were in bold.

**Species**	**Location**	**VoucherNumber**	**GenBank Number**				**References**
			**ITS**	**LSU**	***rpb2***	***tef1-α***	
*Entoloma acutiflavum*	China	HKAS 150127	PX269826	PX269836	PX255550	PX255527	(Liu et al. 2025)
*E. acutiflavum*	China	HKAS 150128 holotype	PX269825	PX269837	PX255549	PX255526	(Liu et al. 2025)
*E. acutipallidum*	Brazil	FK1893	—	MG018325	—	MH190147	(Liu et al. 2025)
*E. acutipallidum*	Brazil	11RMTO78	—	MW624792	MW624730	—	(Karstedt et al. 2024)
*E. acutoconicum*	Papua New Guinea	ZTMyc42856	—	MW624791	—	—	(Karstedt et al. 2024)
*E. albidoquadratum*	India	PM667 holotype	—	GQ289151	GQ289223	—	(Liu et al. 2025)
*E. albogracile*	Papua New Guinea	ZTMyc42855	—	MH190207	—	—	(Liu et al. 2025)
*E. altissimum*	Vietnam	LE262945	MF476912	MW624793	MW624731	—	(Karstedt et al. 2024)
*E. amazonicum*	Brazil	11RMT126	—	MW624794	MW624732	MW624839	(Karstedt et al. 2024)
*E. amazonicum*	Brazil	FK1815 holotype	—	MW624795	—	—	(Karstedt et al. 2024)
*E. arenicola*	Brazil	FK1811 holotype	—	MW624796	MW624733	—	(Karstedt et al. 2024)
*E. arenicola*	Brazil	FK2089	—	MW624797	MW624734	MW624840	(Karstedt et al. 2024)
*E. atropapillatum*	Brazil	FK0898 holotype	KF679354	KF738940	MH190107	MH190137	(Karstedt et al. 2024)
*E. azureoviride*	Brazil	FK1123	—	MW624830	MW624752	MW624855	(Karstedt et al. 2024)
*E. bichromum*	China	HKAS 150132 holotype	PX269813	PX269834	PX255538	PX255530	(Liu et al. 2025)
*E. bichromum*	China	HKAS 150131	PX269814	PX269835	PX255539	PX255531	(Liu et al. 2025)
*E. borbonicum*	France	WU21097 holotype	—	MH190198	MH190131	MH190166	(Liu et al. 2025)
*E. canoconicum*	New Zealand	ZTMyc42846	—	MW624801	—	—	(Karstedt et al. 2024)
*E. canoconicum*	New Zealand	ZTMyc42850	—	MW624802	MW624736	—	(Karstedt et al. 2024)
*E. capes*	Brazil	FK2096 holotype	—	MW624803	MW624737	—	(Karstedt et al. 2024)
*E. caribaeum*	Brazil	FK1790	—	MH190214	MH190114	MH190146	(Liu et al. 2025)
*E. carneum*	Vietnam	LE262912	—	MH190181	MH190119	MH190152	(Liu et al. 2025)
*E. carneum*	Vietnam	LE262954	—	MH190184	MH190121	—	(Liu et al. 2025)
*E. caxiuanense*	Brazil	FK1871 holotype	—	MW624804	—	—	(Karstedt et al. 2024)
*E. cervinum*	Brazil	FK1770	—	MW624805	—	—	(Karstedt et al. 2024)
*E. cycneum*	Vietnam	LE F-343654 holotype	OQ779461	OQ804518	—	OQ779183	(Morozova and Pham 2023)
*E. cycneum*	Vietnam	LE F-343655	OQ779463	OQ804519	—	OQ779182	(Morozova and Pham 2023)
*E. dennisii*	USA	8263 TJB	—	MH190195	MH190128	MH190164	(Liu et al. 2025)
*E. dragoluteum*	Brazil	FK2131 holotype	—	MW624806	MW624738	—	(Karstedt et al. 2024)
*E. dragoluteum*	Brazil	FK2120	—	MW624807	MW624739	MW624842	(Karstedt et al. 2024)
*E. dragonosporm*	Brazil	FK2019	—	MH190179	MG018336	MH190150	(Liu et al. 2025)
*E. dragonosporm*	Brazil	MC4600	—	MH190186	MH190122	MH190156	(Liu et al. 2025)
*E. dragorufescens*	Brazil	FK2102 holotype	—	MW624810	MW624740	MW624843	(Karstedt et al. 2024)
*E. dragorufescens*	Brazil	FK2116	—	MW624811	MW624741	—	(Karstedt et al. 2024)
*E. excavatum*	China	HFJAU2013 holotype	PP796416	PP789602	—	—	(Chen et al. 2024)
*E. excavatum*	China	HFJAU4774	PP796431	PP789614	—	—	(Chen et al. 2024)
***E. fucatum***	**China**	**HFJAU2253**	**PZ242232**	**PZ240433**	**PZ270250**	**PZ270247**	**This work**
***E. fucatum***	**China**	**HFJAU2690 holotype**	**PZ242233**	**PZ240434**	—	—	**This work**
*E. gatesianum*	Brazil	ACM498 holotype	—	MW624812	MW624742	MW624844	(Karstedt et al. 2024)
*E. gatesianum*	Brazil	ACM499	—	MW624813	—	—	(Karstedt et al. 2024)
*E. guttuliferum*	China	HKAS 150129 holotype	PX269815	PX269838	PX255540	PX255528	(Liu et al. 2025)
*E. hochstetteri*	New Zealand	TL2570	KP191939	KP191755	—	—	(Liu et al. 2025)
*E. hochstetteri*	New Zealand	TL2573	KP191941	KP191758	—	—	(Liu et al. 2025)
*E. hochstetteri*	New Zealand	ZTMyc42838	—	MW624814	—	—	(Liu et al. 2025)
*E. hochstetteri*	New Zealand	ZTMyc42841	—	OP836300	—	—	(Liu et al. 2025)
*E. kovalenkoi*	Vietnam	LE312529 holotype	OK257210	OK257207	—	OK256169	(Liu et al. 2025)
*E. kovalenkoi*	Vietnam	LE312530	OK257211	OK257208	—	—	(Chen et al. 2024)
*E. lacticolor*	China	HFJAU3721	OR683791	OR687488	OR738708	OR699449	(Chen et al. 2024)
*E. lacticolor*	China	HFJAU3728	OR683792	OR687489	OR738709	OR699450	(Chen et al. 2024)
*E. lacticolor*	China	HFJAU3736 holotype	OR683793	OR687490	OR738710	OR699451	(Chen et al. 2024)
*E. luteobrunneum*	Brazil	FK1693 holotype	—	MW624816	—	MW624846	(Karstedt et al. 2024)
*E. luteolamellatum*	Brazil	11RMT109	—	MH190170	MH190105	—	(Karstedt et al. 2024)
*E. luteolamellatum*	Brazil	FK1866	—	MW624817	MW624743	—	(Karstedt et al. 2024)
*E. luteum*	China	GDGM 27698	JQ281486	JQ320121	—	—	(Liu et al. 2025)
*E. manausense*	Brazil	ACM500	—	MW624818	MW624744	MW624847	(Karstedt et al. 2024)
*E. manausense*	Brazil	FK2083	—	MW624819	MW624745	—	(Karstedt et al. 2024)
*E. mocamboense*	Brazil	FK1899 holotype	—	MW624820	—	MW624848	(Karstedt et al. 2024)
*E. murrayi*	Switzerland	VHAs0202	—	GU384620	GU384637	—	(Liu et al. 2025)
*E. murrayi*	China	QI 1002	KJ658968	JQ993089	JQ993082	—	(He et al. 2015)
*E. murrayi*	China	QI 1001	KJ658967	JQ993090	JQ993081	—	(He et al. 2015)
*E. murrayi*	China	HKAS 52597	—	KJ648469	—	—	(Liu et al. 2025)
*E. murrayi*	Russia	LE253781	—	MH190180	MH190118	—	(Karstedt et al. 2019)
*E. murrayi*	USA	8210 TJB	—	MH190193	MH190127	MW624849	(Karstedt et al. 2019)
*E. neotropicale*	Brazil	FK2016	—	MW624822	MW624746	MW624850	(Karstedt et al. 2024)
*E. neotropicale*	Brazil	FK2130 holotype	—	MW624825	MW624748	MW624853	(Karstedt et al. 2024)
*E. pallidoflavum*	Vietnam	LE262934	OQ779469	MH190183	MH259314	MH190155	(Karstedt et al. 2019)
*E. paulense*	Brazil	FK0821	—	MW624826	MW624749	—	(Karstedt et al. 2024)
*E. paulense*	Brazil	FK1151 holotype	—	MW624827	MW624750	—	(Karstedt et al. 2024)
*E. percuboideum*	Austria	WU7051	—	MH190200	—	—	(Karstedt et al. 2024)
*E. peristerinum*	Vietnam	LE F-343653 holotype	OQ779466	OQ804522	—	OQ779188	(Morozova and Pham 2023)
*E. peristerinum*	Vietnam	LE F-343650	OQ779467	OQ804524	—	OQ779186	(Morozova and Pham 2023)
***E. pseudobrunneosquamulosum***	**China**	**HFJAU2700 holotype**	**PZ242234**	**PZ240436**	**PZ270251**	**PZ270248**	**This work**
***E. pseudobrunneosquamulosum***	**China**	**HFJAU5732**	**PZ242235**	**PZ240435**	**PZ270252**	**PZ270249**	**This work**
*E. petchii*	China	HKAS 56716	JQ281485	JQ320120	—	—	(Liu et al. 2025)
*E. phlebophyllum*	China	HFJAU4261 holotype	OR827447	OR825714	OR827308	OR827307	(Chen et al. 2024)
*E. phlebophyllum*	China	HFJAU3126	OR827451	OR826040	—	—	(Chen et al. 2024)
*E. plicatum*	Australia	DLL9691	—	JQ624610	JQ624617	—	(Liu et al. 2025)
*E. plicatum*	Australia	DLL10083	—	JQ624612	JQ624619	MG702626	(Liu et al. 2025)
*E. prismaticum*	Japan	K381 holotype	AB691998	AB692006	AB692016	—	(Karstedt et al. 2024)
*E. procerum*	New Zealand	PDD75517	—	MH190189	—	—	(Karstedt et al. 2019)
*E. procerum*	New Zealand	ZTMyc42821	—	MH190201	—	MH190167	(Karstedt et al. 2019)
*E. pseudotomentosum*	China	HFJAU5159 holotype	PP796434	PP789617	PP873253	PP873237	(Chen et al. 2024)
*E. pseudotomentosum*	China	HFJAU5160	PP796435	PP789618	PP873254	PP873238	(Chen et al. 2024)
*E. pseudotomentosum*	China	HFJAU5166	PP796436	PP789619	PP873255	PP873239	(Chen et al. 2024)
*E. quadratum*	USA	EQ7695	—	AF261303	—	—	(Liu et al. 2025)
*E. quadratum*	Russia	LE254355	KC898452	KC898504	—	—	(Liu et al. 2025)
*E. quadratum*	USA	7794 TJB	—	MH190192	MH190126	—	(Liu et al. 2025)
*E. quadratum*	USA	8214 TJB	—	MH190194	—	MH190162	(Liu et al. 2025)
*E. quadratum*	China	HFJAU5173	PP796437	PP789620	PP873256	PP873240	(Chen et al. 2024)
*E. quadratum*	China	HFJAU5179	PP796438	PP789621	PP873257	PP873241	(Chen et al. 2024)
*E. rufomarginatum*	China	HFJAU1933 holotype	PP796415	PP789601	—	—	(Chen et al. 2024)
*E. rufomarginatum*	China	HFJAU4070	PP883966	PP789608	—	—	(Chen et al. 2024)
*E. rufosquamulosum*	China	HKAS 130186 holotype	PX269818	PX269828	PX255543	PX255532	(Liu et al. 2025)
*E. rufosquamulosum*	China	HKAS 150126	PX269819	PX269829	PX255544	PX255533	(Liu et al. 2025)
*E. sericeum*	USA	VHAs03/02	DQ367430	DQ367423	DQ367435	DQ367428	(Liu et al. 2025)
*E. sericeum*	Germany	KaiR237	OL338118	OL338542	OL405220	—	(Reschke et al. 2022)
*E. smurfetti*	Brazil	FK1741 holotype	—	MW624829	MW624751	MW624854	(Karstedt et al. 2024)
*E. smurfetti*	Brazil	FK1709	—	MW624831	—	—	(Karstedt et al. 2024)
*E. subcycneum*	China	HFJAU4738	PP796430	PP789613	—	—	(Chen et al. 2024)
*E. subcycneum*	China	HFJAU3124 holotype	PP796420	—	PP873245	PP873229	(Chen et al. 2024)
*E. submurrayi*	China	HFJAU3587 holotype	PP796423	PP789606	—	PP873230	(Chen et al. 2024)
*E. tenue*	Brazil	FK1922	—	MH190176	MH190115	—	(Karstedt et al. 2024)
*E. virescens*	Australia	MCA2479	—	GU384622	GU384640	MG702629	(Karstedt et al. 2019)
*E. virescens*	Australia	DLL9972	—	KR869937	KR869957	MG702628	(Karstedt et al. 2019)
*E. virescens*	USA	WU21096	—	MH190197	—	—	(Karstedt et al. 2024)
*E. voltavelhense*	Brazil	FK1694 holotype	—	MW624834	MW624753	MW624856	(Karstedt et al. 2024)
*E. voltavelhense*	Brazil	FK2118	—	MW624835	MW624754	—	(Karstedt et al. 2024)

## Results

### Phylogenetic analysis

A total of 276 sequences (47 ITS, 106 LSU, 68 *rpb*2, and 55 *tef*1-α) from 107 samples were used for phylogenetic analyses using BI and ML. A total of 3,975 characters were used in phylogenetic analyses (ITS, 1,409 bp; LSU, 819 bp; *rpb*2, 609 bp; *tef*1-α, 1,138 bp), of which 2,304 were constant, 1,390 were parsimony-informative, and 281 were singletons. The best-fit models of both ML and BI were the same: GTR + F + I + G4 for ITS, GTR + F + I + G4 for LSU, SYM + I + G4 for *rpb*2, and GTR + F + I + G4 for *tef*1-α. For Bayesian analysis, the average standard deviation of split frequencies was less than 0.01 after 1,625,000 generations.

**Figure 1.**
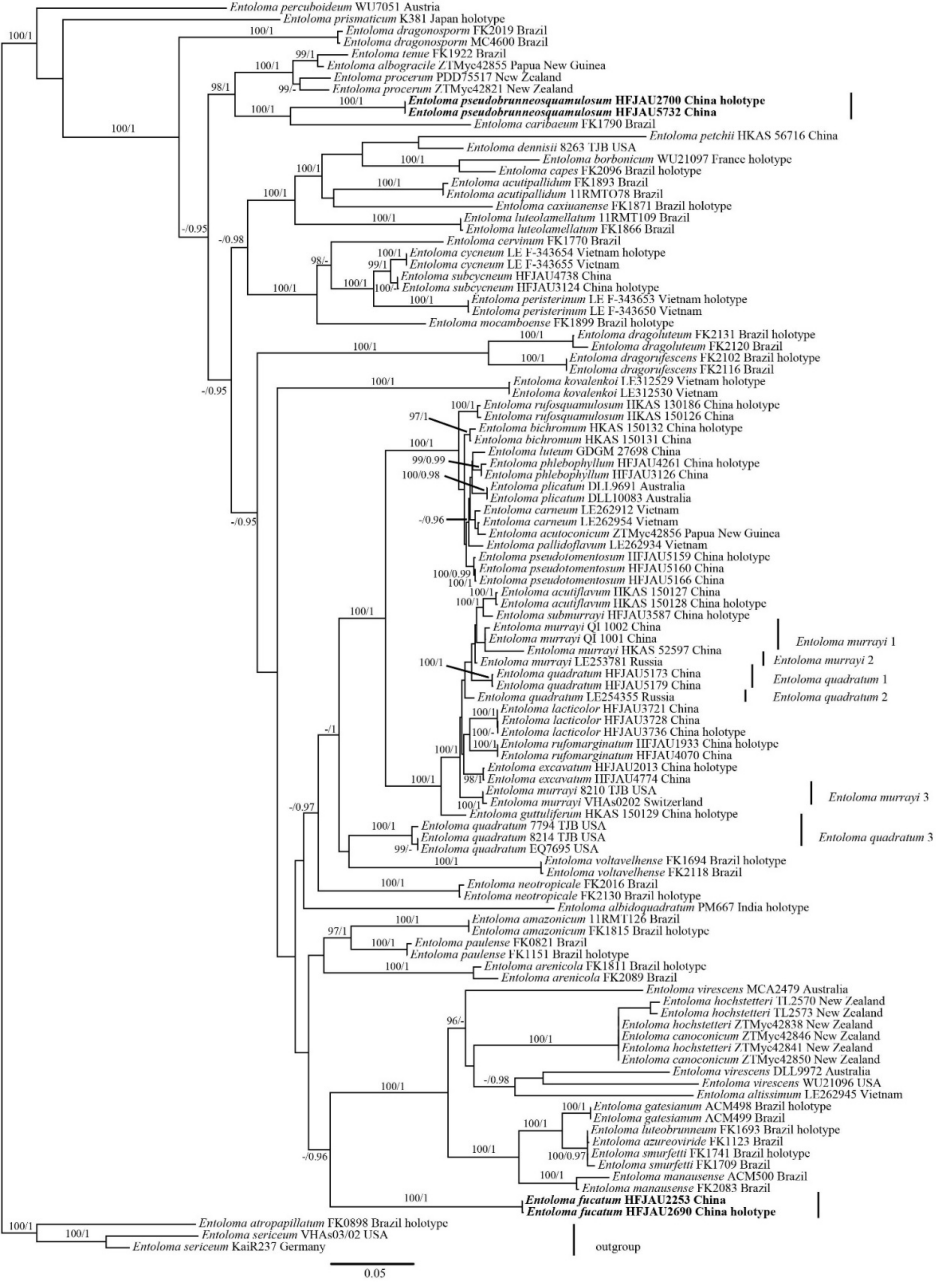
Phylogram of *Entoloma* subgenus *Cubospora* spp. generated by maximum likelihood (ML) analysis based on ITS, LSU, *rpb*2, and *tef*1-α, rooted with *E.* subgenus *Nolanea* spp. Bayesian inference (BI-PP) ≥ 0.95 and ML bootstrap proportions (UFBoot) ≥ 95% are indicated as PP/UFBoot. The new taxa are marked in bold.

The phylogenetic results are shown in Fig. 1. The BI and ML topologies were highly congruent; therefore, the ML tree is presented with Bayesian posterior probabilities (PP) and ultrafast bootstrap support values (UFBoot) indicated at the nodes (Fig. 1). *Entoloma* subg. *Cubospora* formed a well-supported monophyletic clade. The two new taxa, *E. fucatum* and *E. pseudobrunneosquamulosum*, each formed well-supported, distinct lineages within *E.* subg. *Cubospora*. The phylogenetic position of *E. fucatum* is unknown in the subgenus, whereas *E. pseudobrunneosquamulosum* grouped with *E. caribaeum* (Pegler) Courtec. & Fiard.

### Taxonomy

#### Entoloma fucatum

Taxon classificationFungiAgaricalesEntolomataceae

J.Q. Yan, Y.Q. Xu & S.N. Wang sp. nov.

BDCDA16B-7A46-50B8-86D7-0FF92D878C58

863208

Fig. 2

##### Etymology.

Refers to the cosmetic-like, reddish brown coloration of the pileus.

##### Holotype.

**China** • Zhejiang Province, Lishui City, Liandu District, Jun-Qing Yan, Ze-Wei Liu, 4 August 2021, HFJAU2690.

##### Diagnosis.

*Entoloma fucatum* is mainly characterized by the rather small basidiomata; pileus white with a greyish ruby center, hygrophanous, non-striate; basidiospores cuboid; lamella edges sterile; pleurocystidia and cheilocystidia present; and clamp connections present.

##### Macromorphology.

Basidiomata rather small. Pileus 11.0–26.0 mm wide, campanulate to convex with umbonate or slight umbonate center, white, greyish-ruby (12D5) to dark ruby (12F8), darker at center, paler toward margin, the center fades to white when dry, smooth, hygrophanous, not striate, margin entire and involute. Lamellae moderately distant, 2.0–4.0 mm wide, adnate to emarginate, ventricose, with two to three tiers of lamellulae, white to flesh-pink, edge crenate, concolorous. Stipe 25.0–60.0 × 2.0–5.0 mm, central, hollow, terete, slightly tapering towards the apex, concolorous with the pileus, its surface is twisted with grooves, base with white mycelium.

##### Micromorphology.

Basidiospores [80/4/2] (8.0)8.3–10.0(10.5) × (8.0)8.4–10.0(10.2) μm, *av* = 9.1 × 9.2 μm, *Q* = 1.0 (*Qm* = 1.00 ± 0.01), isodiametric, cuboid, sporadically with five angles in side-view, thick-walled, inamyloid. Basidia 35.0–56.5 × 11.0–17.5 μm, clavate, cylindric-clavate, 2-or 1-or 4-spored, clamped. Pleurocystidia scattered, fusoid, lageniform, rarely furcate, 24.0–61.0 × 9.0–20.0 μm. Lamella edge sterile. Cheilocystidia clustered on lamella edge, 22.0–61.5 × 7.5–14.0 μm, clavate, cylindric-clavate. Lamellar trama regular, made up of cylindrical hyphae 5.5–14.0 μm wide. Pileipellis is a cutis to a trichoderm of cylindrical hyphae 4.0–13.0 μm broad, slightly constricted or not constricted at the septa, with rounded or acute end and grayish yellow membranal pigment. Stipitipellis a cutis composed of densely arranged, cylindrical hyphae 4.0–16.5 μm wide, slightly constricted or not constricted at the septa, with rounded or acute end and light brownish yellow membranal pigment. Clamp connections are present in all tissues.

**Figure 2.**
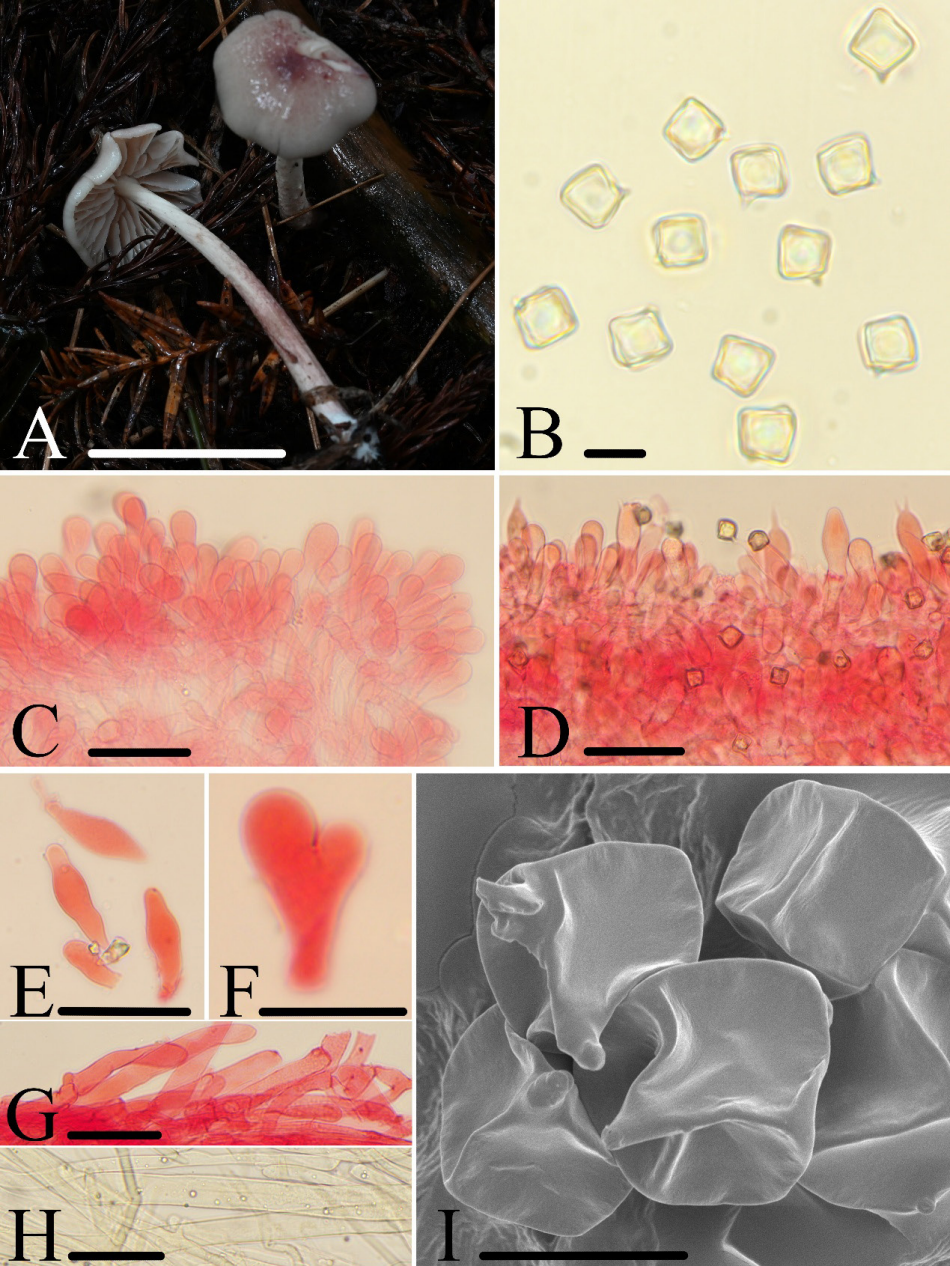
*Entoloma fucatum*: **A**. Basidiomata; **B, I**. Basidiospores; **C**. Cheilocystidia; **D**. Pleurocystidia and Basidia; **E–F**. Pleurocystidia; **G**. Pileipellis; **H**. Stipitipellis. **H** was observed in water, **B–G** were observed in 5% KOH, and 1% Congo red was used as the stain except **B**. Scale bars: 30 mm (**A**); 10 μm (**B, I**); 40 μm (**C**); 50 μm (**D, E**); 20 μm (**F**); 40 μm (**G**); 50 μm (**H**).

##### Habitat.

Scattered on the soil surface in a mixed coniferous-broad-leaved forest dominated by *Cupressaceae* species with a small number of *Fagaceae* plants.

##### Additional specimens examined.

**China** • Zhejiang Province, Lishui City, Suichang County, Yan-Liu Chen, 15 July 2020, HFJAU2253.

##### Notes.

In the phylogenetic tree (Fig. 1), *E. fucatum* showed no close affinity to any known congeneric species. Based on the BLAST alignment results, *E. fucatum* shares 95.69%, 95.82%, and 95.69% similarity of LSU sequences, respectively, with *E. pseudotomentosum* J.Q.Yan, Y.Q.Xu & S.N.Wang, *E. quadratum* (Berk. & M.A.Curtis) E.Horak, and *E. submurrayi* J.Q.Yan, L.G.Chen & S.N.Wang. However, *E. pseudotomentosum* features a yellowish green pileus center covered with scales and lacks pleurocystidia; *E. quadratum* is characterized by an orange-red pileus with striations and lacks pleurocystidia; *E. submurrayi* has a golden-yellow pileus with striations and also lacks pleurocystidia (Chen et al. 2024).

In addition, within this subgenus, only *E. arenicola* Karstedt & Capelari resembles the new species *E. fucatum* in possessing the combined characteristics of red basidiomata with a hygrophanous pileus and cuboid spores, but the former lacks pleurocystidia (Karstedt et al. 2024).

#### Entoloma pseudobrunneosquamulosum

Taxon classificationFungiAgaricalesEntolomataceae

J.Q. Yan, Y.Q. Xu & S.N. Wang sp. nov.

04D58818-F8AB-5397-8AFC-CA15F5A00598

863210

Fig. 3

##### Etymology.

Refers to its morphology similar to “*Entoloma brunneosquamulosum*.”

##### Holotype.

**China** • Zhejiang Province, Lishui City, Qingtian County, 5 August 2021, collected by Jun-Qing Yan, Ze-Wei Liu, HFJAU2700.

##### Diagnosis.

*Entoloma pseudobrunneosquamulosum* is mainly characterized by small, collybioid basidiomata; pileus brown, covered with squamules, non-striate; basidiospores cuboid; lamella edge heterogeneous, with cheilocystidia and pleurocystidia present; pileipellis with brown intracellular pigments.

**Figure 3.**
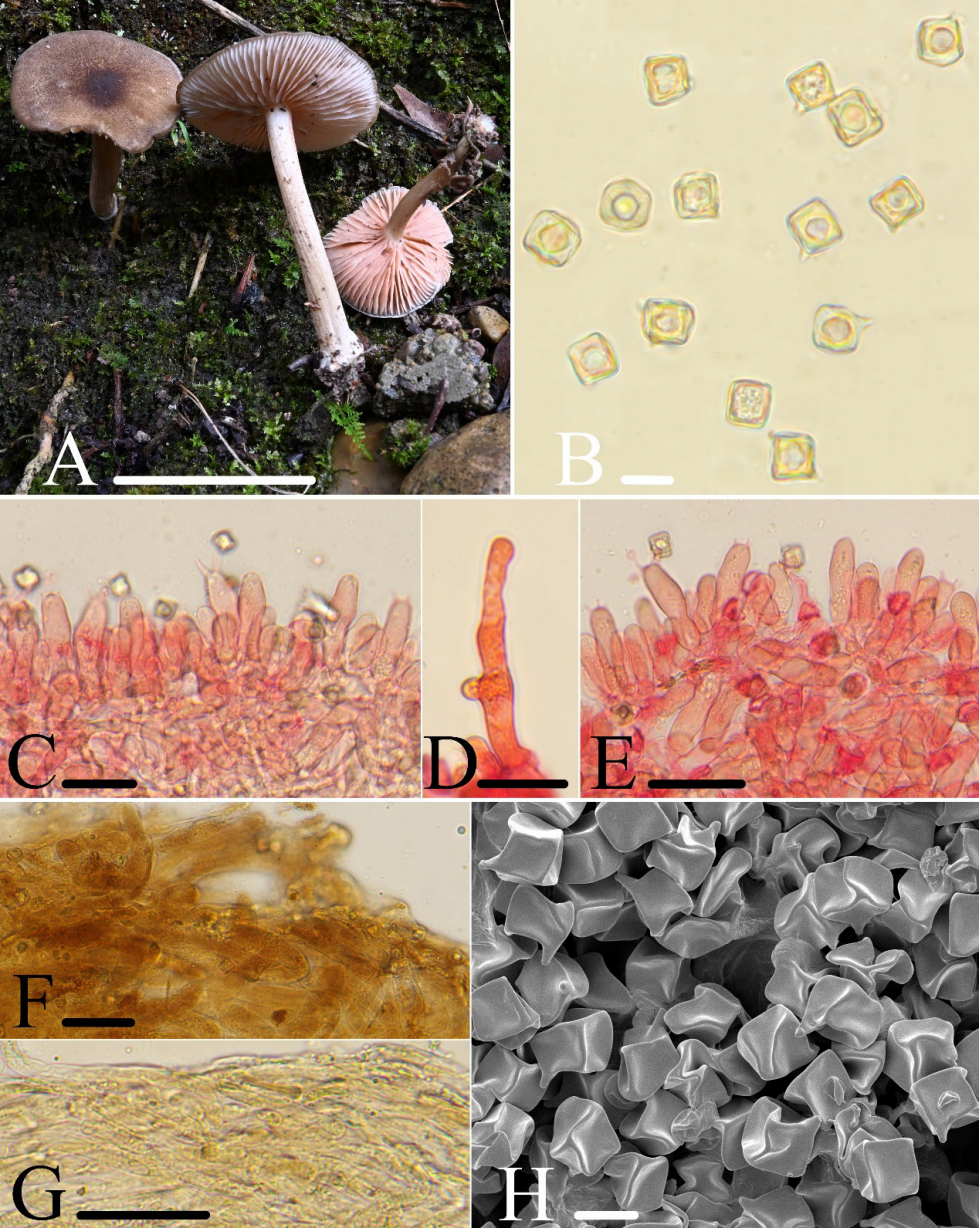
*Entoloma pseudobrunneosquamulosum*: **A**. Basidiomata; **B, H**. Basidiospores; **C**. Heterogeneous lamella edge; **D**. Cylindric cheilocystidia; **E**. Pleurocystidia and Basidia; **F**. Pileipellis; **G**. Stipitipellis. **F, G** were observed in water; **B–E** were observed in 5% KOH, and 1% Congo red was used as the stain except **B**. Scale bars: 40 mm (**A**); 10 μm (**B, H**); 30 μm (**C**); 25 μm (**D**); 40 μm (**E–G**).

##### Macromorphology.

Basidiomata small, collybioid. Pileus 26.0–40.0 mm wide, convex to plano-convex, reddish brown (8E5) to dark brown (8F8), pileus surface brown, covered with hairy scales and non-striate; the pileus margin exceeding the lamellae, margin entire. Lamellae moderately distant, 2.0–4.0 mm wide, adnate, ventricose, with two to three tiers of lamellulae, white or brownish pink, edge entire, concolorous. Stipe 30.0–55.0 × 4.0–7.0 mm, central, terete, hollow, occasionally twisted, equal, concolorous or paler than the pileus, sometimes sulcate, white tomentose at the base.

##### Micromorphology.

Basidiospores [170/4/2] (6.5)7.2–10.2(11.5) × (6.8)7.4–10.2(10.9) μm, *av* = 9.0 × 9.0 μm, *Q* = 1.0 (*Qm* = 1.00 ± 0.01), isodiametric, cuboid, occasionally 5-angled in side-view, thick-walled, inamyloid. Basidia 34.0–50.0 × 10.0–14.0 μm, clavate, cylindric-clavate, 4- or 2-spored. Pleurocystidia scattered, cylindric-clavate, 31.0–49.0 × 8.5–15.0 μm. Lamella edge heterogeneous. Cheilocystidia dispersed on lamella edge, extremely sparse, 31.5–46.0 × 8.5–15.0 μm, cylindric-clavate, lageniform, rarely hyphoid or cylindric, 23.0–63.0 × 5.0–14.0 μm, clamped. Lamellar trama regular, made up of cylindrical hyphae 5.0–14.0 μm wide. Pileipellis is a cutis with transitions to a trichoderm towards the margin, composed of cylindrical hyphae 6.5–19.0 μm broad, slightly constricted or not constricted at the septa, with rounded or acute ends and sepia-brown intracellular pigment. Stipitipellis a cutis composed of densely arranged, cylindrical hyphae 4.5–10.5 μm wide, slightly constricted or not constricted at the septa, with rounded or acute end and light brown membranal pigment. Brilliant granules are abundant and clamp connections are present in all tissues.

##### Habitat.

Scattered on the ground in mixed forests predominantly composed of *Cupressaceae* species, with a small number of *Fagaceae* plants.

##### Additional specimen examined.

**China** • Zhejiang Province, Lishui City, Qingtian County, 5 August 2021, collected by Jun-Qing Yan, Ze-Wei Liu, HFJAU5732.

##### Notes.

In the phylogenetic tree (Fig. 1), *E. pseudobrunneosquamulosum* grouped with *E. caribaeum* (Pegler) Courtec. & Fiard. *E. pseudobrunneosquamulosum* is easily separated from *E. caribaeum* by its brown pileal surface without striations and the presence of pleurocystidia. *E. caribaeum* has a white pileal surface with striations and lacks pleurocystidia (Karstedt et al. 2024). Based on the BLAST alignment results, *E. pseudobrunneosquamulosum* shares 98.16% similarity of LSU sequences with *E. pseudotomentosum* J.Q.Yan, Y.Q.Xu & S.N.Wang, *E. quadratum* (Berk. & M.A.Curtis) E.Horak, and *E. submurrayi* J.Q.Yan, L.G.Chen & S.N.Wang. However, *E. pseudotomentosum* has white basidiomata and lacks pleurocystidia (Chen et al. 2024); *E. quadratum* and *E. submurrayi* have small, mycenoid basidiomata, with a pileus featuring distinct umbos and striations, and lack pleurocystidia (Horak 1976; Chen et al. 2024).

*Entoloma pseudobrunneosquamulosum* is easily confused with *E. heimii* (Romagn.) Eyssart., Buyck & Courtec. and *E. pseudoheimii* Eyssart., Buyck & Courtec., all of which have a brown pileus with squamules and both cheilocystidia and pleurocystidia. However, *E. heimii* is distinguished by significantly longer cheilocystidia (50.0–70.0 μm) and the absence of clamp connections; *E. pseudoheimii* exhibits deeply sinuate to almost free gills, larger basidiospores (11.5–13.0 × 9.5–11.0 μm), and also lacks clamp connections (Eyssartier et al. 2001).

Additionally, *E. pseudobrunneosquamulosum* exhibits morphological similarities to *E. brunneosquamulosum* C.K.Pradeep & K.B.Vrinda, *E. keralense* Manim. & Noordel., and *E. luteolamellatum* (Largent & Aime) Blanco-Dios. All three have a brown pileus with squamules. However, *E. brunneosquamulosum* is distinguished by adnexed gills, a stipe densely covered with squamules, larger basidiospores (13.0–15.0 × 12.0–14.0 μm), and the absence of pleurocystidia (Pradeep et al. 2013); *E. keralense* features cheilocystidia and pleurocystidia that are ovoid to ventricose with rostrate processes, along with caulocystidia (Manimohan et al. 2006); *E. luteolamellatum* lacks pleurocystidia but has caulocystidia (Karstedt et al. 2024).

#### Entoloma pseudotomentosum

Taxon classificationFungiAgaricalesEntolomataceae

J.Q. Yan, Y.Q. Xu & S.N. Wang nom. nov.

B5587888-D1F5-5C7E-A4D8-5751B827B22B

863237

##### Replaced synonym.

*Entoloma tomentosum* J.Q. Yan, L.G. Chen & S.N Wang, in Chen, Ding, Chen, Zeng, Zeng, Wang & Yan, J. Fungi 10(8, no. 594): 21 (2024); *non Entoloma tomentosum* Z.S. Bi, in Bi, Zheng & Li, Acta Mycol. Sin. 5(3): 165 (1986).

##### Holotype.

**China** • Fujian Province, Wuyishan City, Wuyi Mountain, 17 August 2023, collected by Nian-Kai Zeng, Cheng-Feng Nie, Hua-Zhi Qin, Hui Deng, Tian Jiang, and Run-Xiang Zhao, HFJAU5159.

##### Notes.

*Entoloma tomentosum* J.Q.Yan, L.G.Chen & S.N.Wang (Chen et al. 2024) is a later homonym of *E. tomentosum* Z.S. Bi (Bi et al. 1986) and is herein replaced by *E. pseudotomentosum* J.Q. Yan, Y.Q. Xu & S.N. Wang.

## Discussion

Within this subgenus, the new species described in this study enhance the species diversity of this subgenus in China. However, certain species, such as *E. quadratum* and *E. murrayi*, are noteworthy for being scattered across multiple independent lineages rather than forming a single monophyletic branch (Fig. 1). This phenomenon merits further investigation, as some of these lineages may represent overlooked new species.

This study is based on the investigation of species diversity conducted in subtropical regions. The research findings reveal that the species within the subgenus *Cubospora* are relatively abundant in this area, a viewpoint also corroborated by previous studies (He et al. 2015; Chen et al. 2024; Liu et al. 2025). Therefore, it is necessary to carry out more in-depth investigations and research in this region, with the prospect of clarifying the phylogenetic relationships of this subgenus in the future.

## Supplementary Material

XML Treatment for Entoloma fucatum

XML Treatment for Entoloma pseudobrunneosquamulosum

XML Treatment for Entoloma pseudotomentosum

## Additional information

### Artificial Intelligence (AI) use

The authors accept full responsibility for the content of the manuscript, including the disclosure of any use of AI.

No AI tools were used in the preparation of this manuscript.

### Author ORCIDs

Y-Q. Xu https://orcid.org/0009-0002-0735-8323

H. Zeng https://orcid.org/0000-0003-2025-844X

S-N. Wang https://orcid.org/0000-0003-0648-271X

J-Q. Yan https://orcid.org/0000-0003-1128-5171

### Data availability

All of the data that support the findings of this study are available in the main text or Supplementary Information.
